# *Leishmania donovani* depletes labile iron pool to exploit iron uptake capacity of macrophage for its intracellular growth

**DOI:** 10.1111/j.1462-5822.2008.01241.x

**Published:** 2008-10-09

**Authors:** Nupur Kanti Das, Sudipta Biswas, Sunil Solanki, Chinmay K Mukhopadhyay

**Affiliations:** Special Centre for Molecular Medicine, Jawaharlal Nehru UniversityNew Delhi-110 067, India

## Abstract

Intracellular pathogens employ several strategies for iron acquisition from host macrophages for survival and growth, whereas macrophage resists infection by actively sequestering iron. Here, we show that instead of allowing macrophage to sequester iron, protozoan parasite *Leishmania donovani* (LD) uses a novel strategy to manipulate iron uptake mechanisms of the host and utilizes the taken up iron for its intracellular growth. To do so, intracellular LD directly scavenges iron from labile iron pool of macrophages. Depleted labile iron pool activates iron sensors iron-regulatory proteins IRP1 and IRP2. IRPs then bind to iron-responsive elements present in the 3′ UTR of iron uptake gene transferrin receptor 1 by a post-transcriptional mRNA stability mechanism. Increased iron-responsive element–IRP interaction and transferrin receptor 1 expressions in spleen-derived macrophages from LD-infected mice confirm that LD employs similar mechanism to acquire iron during infection into mammalian hosts. Increased intracellular LD growth by holo-transferrin supplementation and inhibited growth by iron chelator treatment confirm the significance of this modulated iron uptake pathway of host in favour of the parasite.

## Introduction

Intracellular pathogens including bacteria, parasite and viruses avoid initial oxidative challenges of the host macrophage and make their own niches within it (Schaible and Kaufmann, 2004). One of the major challenges they face is to acquire iron that is crucial for their intracellular survival and growth. Thus, these pathogens employ special strategies to acquire iron from macrophages. In contrast, macrophages sequester iron to avoid iron acquisition by invading organisms using different mechanisms like reducing expression of principal iron uptake protein transferrin receptor-1 (TfR1) (Mulero and Brock, 1999), increasing synthesis of iron storage protein ferritin (Drakesmith *et al*., 2005) or by increasing expression of iron release gene ferroportin (Nairaz *et al*., 2007). They also need to protect iron from intracellular pathogens to maintain their own homeostasis, as iron is required as cofactors in many enzymes including those generating reactive oxygen or nitrogen species as immediate defence against invading organisms. Moreover, the macrophages play critical role in body iron homeostasis by recycling iron for erythropoiesis (Andrews and Schmidt, 2007). So, protecting iron from invading organisms is immensely important for macrophage.

The trypanosomatid parasite *Leishmania donovani* (LD) causes splenomegaly and hepatomegaly leading to fatal visceral leishmaniasis in mammalian hosts. After successful entry into macrophages, the promastigote form of parasite proliferates within the mature phagolysosome compartment as amastigote, multiplies within and finally bursts the host to infect neighbouring macrophages (McConville *et al*., 2007). Like other organisms, *Leishmania* also needs to acquire iron from the harsh environment of host macrophages for their intracellular growth (Huynh *et al*., 2006; Marquis and Gros, 2007). The iron acquisition is of further importance for them to avoid oxidative assault of the host, as iron is the cofactor for antioxidant enzyme superoxide dismutase (Fe-SOD) (Paramchuk *et al*., 1997). The inactivation of Fe-SOD affects their virulence and intracellular survival (Ghosh *et al*., 2003). Moreover, intracellular iron status of *Leishmania* is reported to influence their drug-resistance ability (Wong and Chow, 2006). Thus, strategy of iron acquisition from host macrophage is very important for *Leishmania*, which has not been explored much except the recent identification of ZIP family iron transporter LIT1 in *Leishmania amazonensis* expressing in its amastigote form (Huynh *et al*., 2006). However, the source(s) of available iron as well as effect of *Leishmania* infection on iron homeostasis of host macrophage remained largely unexplored.

Macrophage acquires iron via phagocytosed senescent erythrocytes as well as using ubiquitous transferrin (Tf)–TfR1 pathway (Theurl *et al*., 2005). In iron depletion when intracellular labile iron pool (LIP) is decreased, iron-regulatory proteins IRP1 and IRP2 are activated to bind iron-responsive elements (IRE) to upregulate TfR1 by post-transcriptional mRNA stability mechanism (Hentze and Kuhn, 1996). Then TfR1-mediated up-taken iron enriches cytoplasmic LIP for cellular utilization. In this study, we demonstrate that instead of allowing macrophages to sequester iron, virulent LD directly scavenges iron from host LIP to activate IRP–IRE interaction to upregulate TfR1 of host macrophages *in vitro* and *in vivo* and exploits the resultant increase in intracellular iron for its growth.

## Results

### Increased TfR1 expression in macrophages by LD infection

**Fig. 1:**
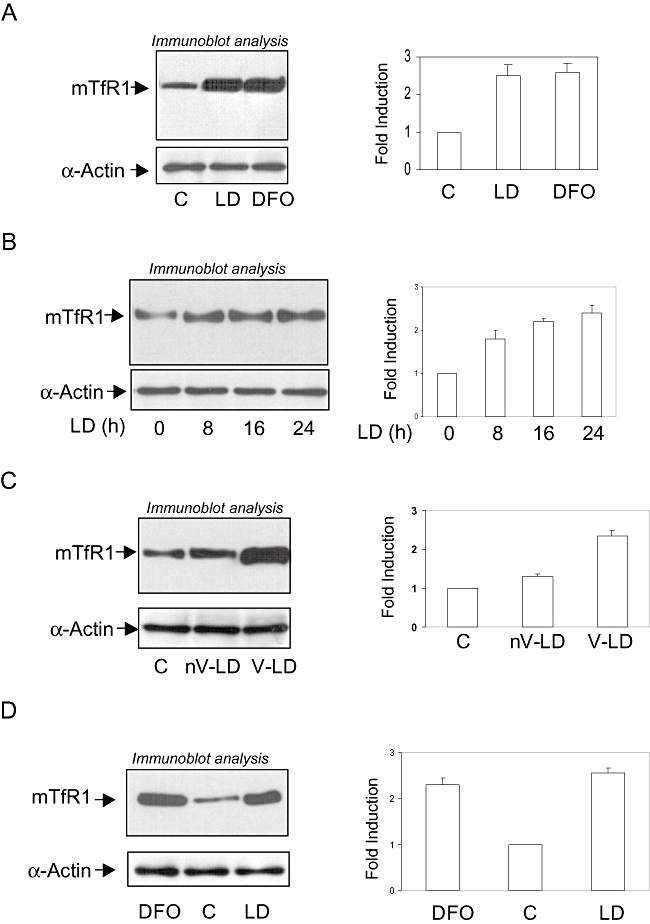
TfR1 expression in macrophages by LD infection. A. J774A.1 cells were infected with freshly isolated virulent LD (1:10) or treated with DFO (100 μM) for 12 h and Western blot analyses were performed with TfR1 (upper panel) or α-actin (lower panel) antibody. Densitometric analysis was shown in the side panel. B. Similarly, Western analyses for TfR1 (upper panel) and α-actin (lower panel) were performed in cell lysates after 0, 8, 16 and 24 h of infection. Relative expressions were determined by densitometric analysis (side panel). C. J774A.1 cells were infected with laboratory-maintained non-virulent LD (nV-LD) and freshly isolated virulent LD (V-LD) for 12 h and Western blot analyses were performed with TfR1 (upper panel) or α-actin (lower panel) antibody. Densitometric analysis was shown in the side panel. D. Macrophages isolated from spleen of uninfected mice were infected with LD for 12 h (10 multiplicity of infection) and Western analyses were performed with TfR1 antibody (upper panel), α-actin (lower panel) and densitometric analysis was shown in side panel. Results are represented as the mean of three observations and normalized to control, arbitrarily chosen as one unit.

To determine the effect of LD infection in macrophages on TfR1 synthesis, J774A.1 cells were infected with freshly transformed virulent LD in a ratio of 1:10 as macrophage : LD. After 12 h of infection TfR1 status was determined in cell lysates by Western analysis. Like iron chelator deferrioxamine (DFO), LD infection also results in a strong induction (2.6-fold) of TfR1 synthesis (Fig. 1A). The induction (1.8-fold) was observed within 8 h and upregulation was detected even after 24 h of infection (Fig. 1B). The increase in TfR1 by LD depends on virulence, as laboratory-maintained non-virulent strain caused a little effect on TfR1 expression (Fig. 1C). We observed that during continuation of LD culture after fresh passage the parasite slowly loses its ability to infect J774A.1 When the infection into J774A.1 in average is more than or equal to three LD infection/macrophage, the increase in TfR1 is detectable, whereas less than three LD infection/macrophage shows marginal or no increase in TfR1 expression (nV-LD, Fig. 1C). In case of more than or equal to five LD infection/macrophage, a strong increase in the TfR1 expression was observed (v-LD, Fig. 1C). If the non-virulent strain is able to cause an infection in mice, then after fresh passage it gains back its capacity to increase TfR1 expression. Similarly, by increasing ratio of non-virulent LD to macrophages, if an average of three or more LD could infect one macrophage, the TfR1 expression is detectable. When macrophages isolated from the spleen of normal Balb/c mice were infected with virulent LD, a 2.7-fold increase in TfR1 expression was detected (Fig. 1D), suggesting that LD could increase TfR1 expression both in cell-cultured and normal macrophages.

### Increased TfR1 expression is due to mRNA stability

**Fig. 2:**
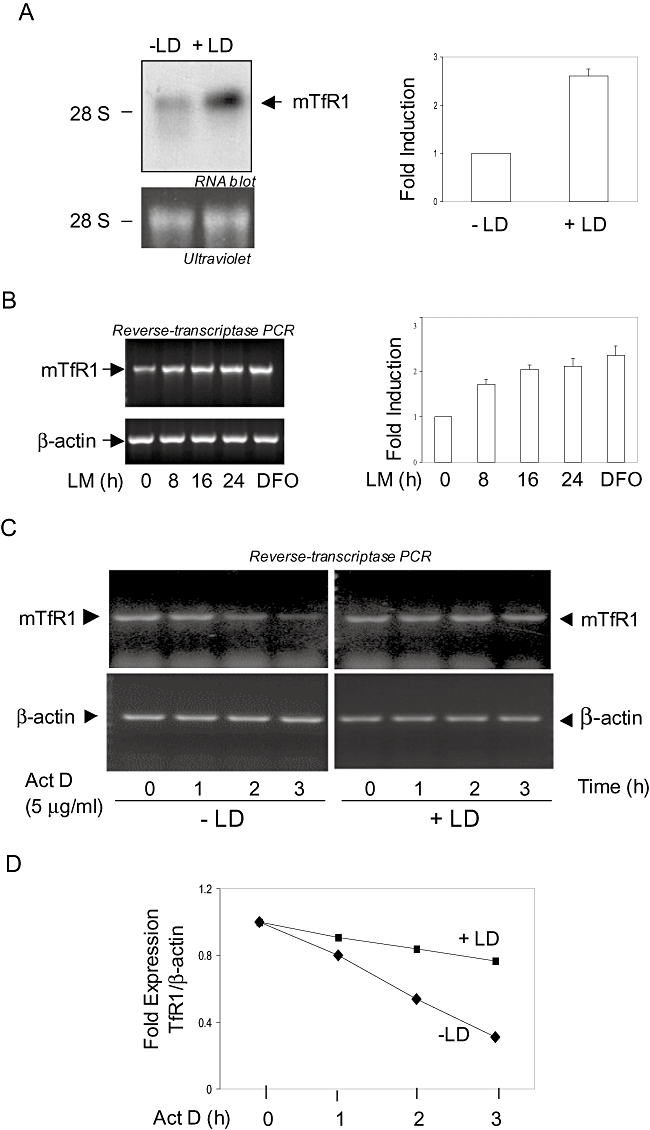
*Leishmania* increases TfR1 synthesis in macrophages by modulating mRNA expression. A. Total RNA was isolated after 10 h of LD-infected J774A.1 cells (10 multiplicity of infection) and abundance of TfR1 mRNA was determined after hybridizing with ^32^P-labelled mouse TfR1 cDNA (upper panel). The abundance of loaded RNA was shown by ultraviolet (lower panel). Relative expression was determined by densitometric analysis (side panel). B. Similarly, total RNA was isolated from *Leishmania major* (LM)-infected J774A.1 cells after 0, 8, 16 and 24 h and RT-PCR was performed with specific primers of mouse TfR1 and β-actin. DFO (100 μM) was used as positive control. Relative expression was determined by densitometric analysis (side panel). C. To determine the mRNA stability of TfR1, uninfected and LD-infected J774 cells were added with actinomycin D (5 μg ml^−1^) after 6 h of infection. Total RNA was isolated at 0, 1, 2 and 3 h of actinomycin D addition and RT-PCR was performed with specific primers of mouse TfR1 and β-actin. D. The relative stability of TfR1 mRNA in LD-infected and uninfected cells was determined by densitometric analysis. Results are represented as the mean of three observations and normalized to control, arbitrarily chosen as one unit.

To understand the mechanism we initially determined TfR1 mRNA expression in LD-infected J774A.1 cells. After 12 h of infection about 2.7-fold induction of TfR1 transcript was detected (Fig. 2A). A time-dependent increase in TfR1 mRNA was also detected by *Leishmania major* (Friedlin) infection into J774A.1 cells as found by semiquantitative reverse transcriptase-PCR (RT-PCR) (Fig. 2B). In iron depletion TfR1 is regulated by mRNA stability mechanism (Hentze and Kuhn, 1996). To find whether a similar mechanism is involved in LD-infected induction of TfR1 expression, mRNA stability assay was performed. Both the LD-infected and uninfected J774A.1 cells were added with transcriptional inhibitor actinomycin D (5 μg ml^−1^) after 6 h of infection. Total RNA was isolated at 0, 1, 2 and 3 h after actinomycin D addition and RT-PCR was performed using mouse TfR1-specific primers. The TfR1 transcript from LD-infected cells was more stable than uninfected control (Fig. 2C). The half-life of TfR1 mRNA from uninfected control (∼2.1 h) is in good agreement with the previous observation (Taetle *et al*., 1987), whereas for infected cells the half-life increased significantly (Fig. 2D).

### LD infection increased IRE–IRP interaction in J774A.1 macrophages

**Fig. 3:**
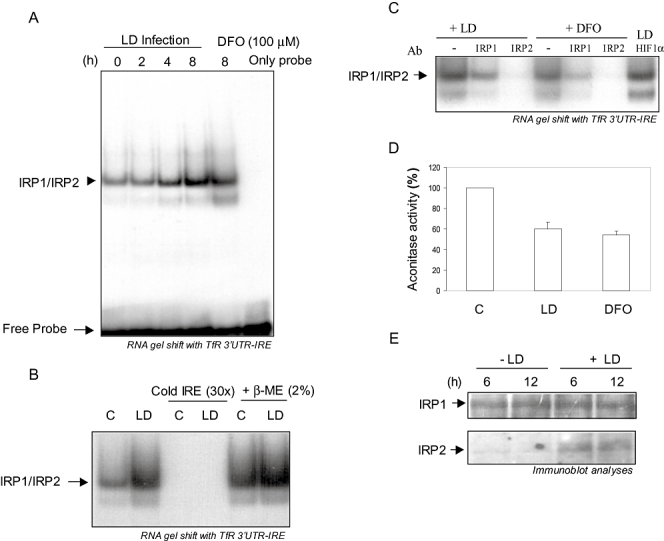
A. RNA gel-shift analysis was performed with *in vitro* transcribed gel-purified ^32^P-labelled IRE containing transcript with cytosolic extracts isolated from J774A.1 macrophages after 0, 2, 4 and 8 h of infection with LD (10 multiplicity of infection). DFO (100 μM)-treated cytosolic extract was used as a positive control. Only probe without the incubation of cytosolic extract was run in the last lane. B. To test the specificity of the IRP–IRE interaction radiolabelled probe was incubated with 30× molar excess of unlabelled purified probe (lanes 3 and 4) or cytosolic extracts were incubated with 2% β-ME prior to incubation with radiolabelled probe and then gel-shift analysis was performed. C. Either IRP1, IRP2 or HIF-1α antibody (2 μg) was incubated for 30 min with the cytosolic extracts isolated from LD-infected or DFO (100 μM)-treated J774A.1 cells before the addition of radiolabelled IRE probe and gel-shift analysis was performed. D. Cytosolic extracts were isolated from LD-infected or uninfected J774 macrophages after 12 h of infection or DFO (100 μM) treatment and aconitase assay was performed. E. Expressions of IRP1 and IRP2 by LD infection were detected by Western analysis of cytosolic extracts isolated from J774A.1 macrophages after 6 and 12 h of infection. Results are represented as the mean of three observations.

The TfR1 mRNA is usually stabilized due to the interactions of IRPs (IRP1 and IRP2) with IREs present in the 3′UTR of the transcript that protect mRNA from endonucleolytic cleavage (Hentze and Kuhn, 1996). To determine the effect of LD infection on IRE–IRP interaction, initially a 188-nucleotide-long fragment of mouse TfR1 3′UTR containing two proximal IREs was *in vitro* transcribed and ^32^P-labelled IRE containing RNA probe was incubated with cytosolic extracts from J774 macrophages. Cytosolic extracts were isolated after 0, 2, 4 and 8 h of LD infection or 8 h of DFO treatment. RNA gel-shift analysis (Fig. 3A) shows a significant increase in RNA binding activity with the increased time of infection. Iron chelator DFO also increased the IRE–IRP interaction with an identical mobility. The interaction was competed out with 30× molar excess of cold RNA probe (Fig. 3B, lanes 3 and 4). β-Mercaptoethanol (2% β-ME) treatment converts latent IRP into fully active IRP so that the regulation of IRP–IRE interaction is lost (Hentze *et al*., 1989). The increased binding capacity of normal cell cytosolic extract in presence of β-ME (2%) (Fig. 3B, lane 5) strongly indicated the interaction as IRE–IRP. Mammalian cells have two IRPs, IRP1 and IRP2 (Hentze and Kuhn, 1996). To further validate the role of these IRPs we have performed supershift analysis. In presence of IRP1 antibody, the interaction between IRE–IRP was reduced but almost completely blocked with IRP2 antibody, whereas the interaction was not affected in presence of HIF-1α antibody (Fig. 3C). A similar result was also obtained with the iron chelator DFO (positive control). This experiment further confirmed role of IRPs in TfR1 expression in LD-infected macrophage. IRP1 having aconitase activity is regulated post-translationally (Rouault, 2006), whereas higher amount of IRP2 was detected in iron depletion (Iwai *et al*., 1995). LD infection for 12 h decreased about 40% of cytosolic aconitase activity, whereas 45–50% decrease was detected by DFO (Fig. 3D). Detection of unaltered IRP1 expression (Fig. 3E, upper panel) but increased IRP2 expression by Western analysis (Fig. 3E, lower panel) indicates an iron-depleted condition in LD-infected host macrophages.

### LD depletes LIP in host macrophages

**Fig. 4:**
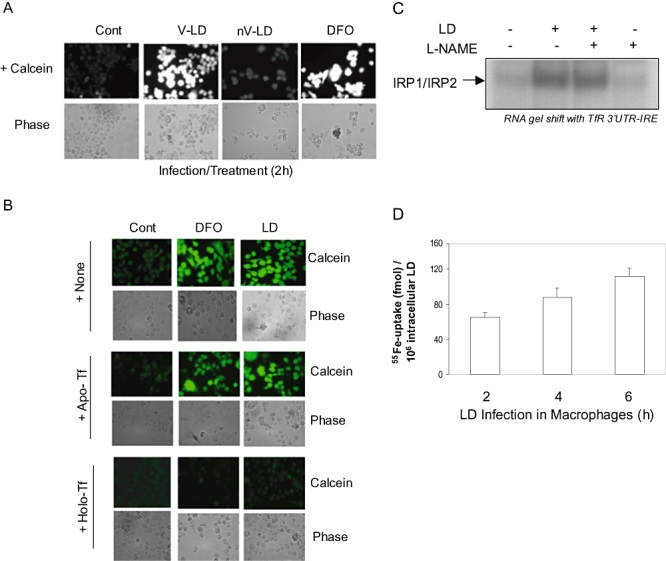
Detection of labile iron pool in host macrophages by LD infection. A. J774A.1 macrophages were infected with either LD (V-LD), non-virulent LD (nV-LD) or incubated with DFO (100 μM) for 2 h and then incubated with calcein-AM (0.5 μM). After 20 min fluorescence level was detected using a microscope (upper panel). Phase contrast pictures are shown in the bottom panel. B. Specificity of detection of LIP was tested by addition of apo-Tf (10 μM) and holo-Tf (10 μM) after 4 h of infection with LD or DFO treatment. C. Cytosolic extracts were prepared from L-NAME (1 mM)-pretreated (1 h) LD-infected J774A.1 cells and gel-shift analysis was performed using radiolabelled IRE probe as described earlier. D. ^55^Fe uptake in intracellular LD from host J774A.1 was determined. Cells were initially incubated with ^55^Fe–Tf for 6 h, washed to remove excess of ^55^Fe–Tf and then infected with LD. After 30 min of infection excess LD was removed and fresh media were added. Then intracellular parasites were isolated at 2, 4 and 6 h of infection from macrophages and ^55^Fe was detected in isolated LD in a scintillation counter. Results are represented as the mean of three observations.

To confirm the possibility of iron depletion in the J774A.1 cell as mechanism of increased IRE–IRP interaction in LD infection we determined LIP using fluorescence indicator dye calcein-AM, which does not show any fluorescence in iron-sufficient condition but fluorescence level is increased in deficiency (Stäubli and Boelsterli, 1998). After 2 h of LD infection calcein-fluorescence was detected significantly higher than uninfected cells (Fig. 4A, panel 2), which could be detected very prominently even after 12 h of infection (data not shown). Laboratory-maintained non-virulent LD strain (nV-LD) showed no significant changes in calcein-mediated fluorescence (Fig. 4A, panel 3), validating our earlier observation of its inability to induce TfR1 synthesis. To confirm that the increase in calcein-fluorescence was only due to intracellular iron depletion, LD-infected cells were supplemented with apo-transferrin (Apo-Tf, 10 μM) or holo-transferrin (Holo-Tf, 10 μM) after 4 h of infection and fluorescence was detected after another 4 h. The complete quenching of fluorescence by holo-Tf but not by apo-Tf (Fig. 4B) confirmed that increased fluorescence was specifically due to decrease in LIP. The IRE–IRP interaction is also known to be increased due to nitric oxide generation (Hentze and Kuhn, 1996). *Leishmania* parasites are well known to suppress nitric oxide generation during infection into macrophages (Forget *et al*., 2006); still to rule out the possibility of any role of nitric oxide in increased IRE–IRP interaction J774A.1 cells were treated with L-NAME, a specific inhibitor of nitric oxide synthase before LD infection. No inhibition of IRE–IRP interaction by L-NAME confirmed that nitric oxide has no influence in increased IRE–IRP interaction in LD-infected macrophage (Fig. 4C). We hypothesized that virulent LD after infection into macrophages increased its own iron uptake capacity to scavenge iron from host LIP. To confirm that LIP of J774A.1 cells was enriched with ^55^Fe before LD infection by incubating with ^55^Fe–Tf, then intracellular LD was isolated from infected J774A.1 in a time-dependent manner up to 6 h and ^55^Fe was determined in isolated LD. Within 2 h of infection a significant amount of ^55^Fe was detected in intracellular parasite, which was increased about twofold after 6 h (Fig. 4D). These results reveal that LD after infection (< 2 h) in macrophages activates its own iron uptake mechanism to scavenge iron from host LIP.

### LD infection in Balb/C mice increases IRP–IRE interaction and TfR1 synthesis in splenic macrophages

**Fig. 5:**
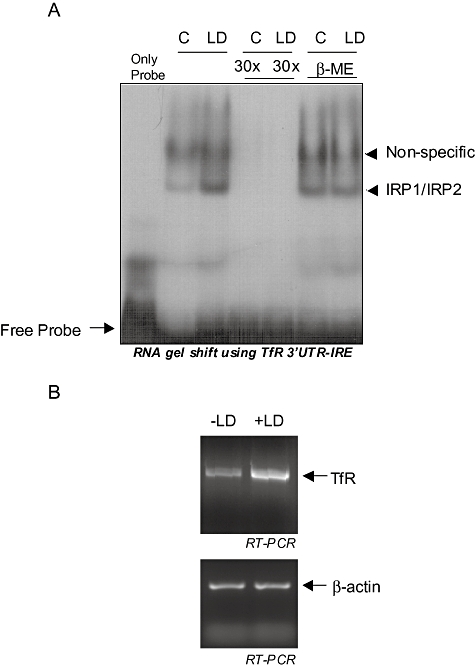
Alteration of iron uptake mechanism in spleen-derived macrophages in LD-infected Balb/c mice. A. One set of Balb/c mice (*n* = 3) were infected with LD and another set were kept uninfected in the similar condition. After 6 weeks splenic macrophages were isolated from both the groups, cytosolic extracts were prepared and RNA gel shift analysis was performed. To check the specificity 30× molar excess of cold probes (lanes 4 and 5) as well as 2% β-ME (lanes 6 and 7) were added with radiolabelled IRE containing *in vitro* transcribed probe. B. Similarly, total RNA was isolated from splenic macrophages of LD-infected and uninfected mice and RT-PCR was performed with primers specific to TfR1 (upper panel) and β-actin (lower panel). Results are representative as one of three independent experiments.

To find whether a similar IRE–IRP interaction and resultant increase in TfR1 expression also happen during LD infection *in vivo*, Balb/C mice were infected with LD for 6 weeks and macrophages were isolated from spleen. When cytosolic extracts from LD-infected and uninfected macrophages were incubated with ^32^P-labelled IRE-containing transcript, an RNA binding activity was observed, which was strongly enhanced in cytosolic extracts isolated from LD-infected spleen-derived macrophages (Fig. 5A). The binding was competed out by 30× molar excess of unlabelled RNA, whereas treatment with 2% β-ME affected the specific RNA binding activity but showed no effect on other non-specific interactions, confirming the increased IRE–IRP interaction in spleen-derived macrophages of LD-infected mice (Fig. 5A). Similarly, increased TfR1 transcript was detected in spleen-derived macrophages from LD-infected Balb/C mice using RT-PCR (Fig. 5B), confirming that LD depletes cellular iron content to activate IRE–IRP interaction to influence macrophage iron uptake mechanism *in vivo*.

### LD utilizes increased intracellular iron of host macrophages for its intracellular growth

**Fig. 6:**
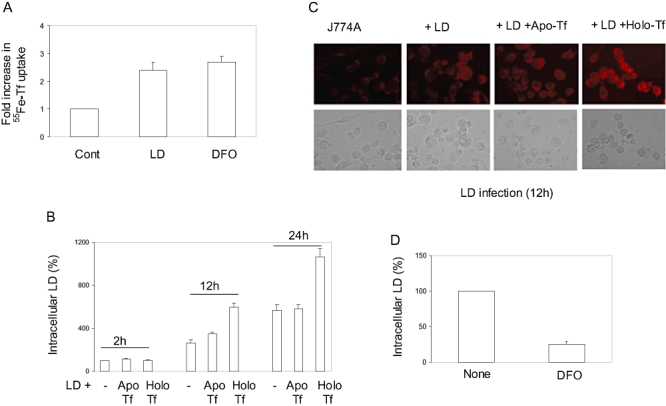
Increased iron uptake by LD-infected macrophages influences intracellular parasite growth. A. J774A.1 macrophages were infected for 12 h with virulent LD or kept uninfected, and then ^55^Fe–Tf was added to both the groups. After 30 min the intracellular ^55^Fe was estimated using cell lysate in a scintillation counter. DFO (100 μM)-treated cells were used as positive control. B. LD-infected J774A.1 cells were supplemented after 4 h of infection either with holo-Tf (10 μM), apo-Tf (10 μM) or none. Total intracellular LD was counted after 2, 12 and 24 h of Tf addition. *P* < 0.001, anova. C. J774A.1 cells were infected with LD and supplemented with Tf like (B), washed, fixed, permeabilized and immunofluoroscence was performed using mAb to LAMP1 after 12 h. D. J774A.1 macrophages were initially treated with DFO (100 μM) for 4 h or none, washed thoroughly to remove DFO and infected with LD. Intracellular LD was counted after 12 h of infection. Results are represented as the mean of three observations and normalized to control.

We hypothesized that LD is utilizing infection-induced increase in intracellular iron in host macrophage for its maintenance and intracellular growth. To verify we initially determined TfR1-mediated iron uptake in LD-infected J774A.1 cells. In presence of ^55^Fe–Tf about 2.5-fold increase in ^55^Fe uptake was observed in LD-infected macrophages compared with uninfected cells (Fig. 6A). To find the significance of this increased intracellular iron, LD-infected cells were supplemented either with physiological concentration of holo-Tf (10 μM), apo-Tf (10 μM) or none and growths of LD were compared. After 2 h of infection the numbers of intracellular parasite of all the groups were similar but after 12 h of infection growth of LD in holo-Tf-supplied J774A.1 was found 2.2-fold higher than other groups, which continued to grow faster at least up to 24 h (Fig. 6B). A remarkable feature of the intracellular LD is to thrive within hostile environment of lysosome. Increased number of intracellular LD would thus cause increased phagolysosome formation, which could be detected by the lysosomal glycoprotein Lamp-1 expression (Huynh *et al*., 2006). In a replica of previous experiment, we detected Lamp-1 expression by fluorescence microscopy. The increased expression of Lamp-1 in holo-Tf-supplemented LD-infected macrophages compared with either apo-Tf or no supplement further confirms increased rate of intracellular LD growth in holo-Tf-supplied macrophages. The growth of only LD-infected and apo-Tf-supplied group was continued probably because of available iron from already existing host LIP. So, when host LIP was depleted by DFO treatment the intracellular growth of LD was reduced by more than 75% (Fig. 6D), which further confirmed the ability of intracellular LD to utilize LIP of host.

## Discussion

One of the hallmark survival strategies of parasites is their ability to control and use the host machineries for their own benefit. Acquiring iron is essential for survival and growth of all the organisms including pathogens residing within macrophages. *Leishmania* spp. also needs to acquire iron from mammalian host for their growth and for effective defence against macrophage's oxidative assault by providing iron to antioxidant enzyme SOD (Ghosh *et al*., 2003). In this study we detected an active mechanism of intracellular LD by which it directly scavenges iron from LIP to activate iron-sensing proteins. IRPs then interact with IREs of TfR1 to increase host iron uptake so that LD could use the iron for its intracellular growth. Thus, unlike responding to most of the pathogens by sequestering iron to avoid infection, macrophages assist growth of intracellular LD. We have also detected a similar ability of *L. major* to increase TfR1 mRNA expression in host J774A.1 cells (Fig. 2B). This novel finding using J774A.1 macrophages was further confirmed in an animal model. Macrophages isolated from spleen of LD-infected Balb/c mice showed increased IRE–IRP interaction (Fig. 5A) and resultant increased transcript of TfR1 (Fig. 5B). To our knowledge this is the first demonstration of increased IRE–IRP interaction for any infection *in vivo.*

We also revealed that LIP is the immediate iron source for intraphagosomal LD. Our experiments confirmed that calcein-mediated fluorescence in LD-infected macrophage was only due to depletion of LIP as complete reversal of increased fluorescence was observed by holo-Tf but not by apo-Tf (Fig. 4B). The intracellular iron depletion is sensed by IRPs. Among the two IRPs, IRP1 is ubiquitously expressed bifunctional protein that acts as a cytosolic aconitase in iron sufficiency but in iron depletion acts as an RNA-binding protein involved in post-transcriptional regulation of iron metabolism (Hentze and Kuhn, 1996). In iron deficiency IRP1 expression remains unaltered but aconitase activity is reduced due to loss of iron (4 Fe to 3 Fe), whereas the IRP2 expression is increased (Iwai *et al*., 1995). During LD infection into macrophages we detected decrease in aconitase activity (Fig. 3D) and increased synthesis of IRP2 (Fig. 3E), suggesting depletion of LIP during LD infection. We also ruled out the possibility of any involvement of nitric oxide in increased IRE–IRP interaction (Fig. 4C). The ability of intracellular LD to scavenge iron was finally confirmed by time-dependent acquisition of iron (^55^Fe) from radiolabelled LIP of macrophages (Fig. 4D). Whether direct scavenging of host LIP and subsequent controlling of macrophage's iron uptake process is unique to *Leishmania* or being used by other intracellular pathogens is the subject of further study, but certainly use of LIP as intracellular iron source provides several advantages to LD. First, acquiring iron from LIP is easier than other intracellular iron store like ferritin because LIP represents an apparently free cytoplasmic iron pool (Andrews, 2004; Theurl *et al*., 2005). Second, decrease of LIP activates IRP1 and IRP2 to increase TfR1 expression and iron uptake. This process should perpetuate the supply of iron within the infected cells. That TfR1 remained upregulated at least up to 24 h (Fig. 1B) suggests that LD scavenges and utilizes iron continuously from LIP. Third, the activation of IRE–IRP interaction should also stop the translation of storage protein ferritin so that host could not sequester LIP into ferritin as a defence strategy. When we determined ferritin expression in LD-infected J774A.1 cells, there was no increase in ferritin as detected by Western analysis (data not shown). Thus, by depleting LIP to increase IRE–IRP interaction, LD actively blocks the host iron sequestration mechanisms.

Interestingly, in a previous study, Tf was suggested to be the iron source for *Leishmania* species like *L. amazonensis* or *L. pifanoi* (Borges *et al*., 1998) because gold-labelled Tf in parasitophorus vacuoles (PV) was detected after 7 days of infection. Originally, the study described that the *Leishmania* could utilize Tf by subverting it from endosome into PV. The study neither described the iron source of the parasite for first 7 days of infection nor tested any regulation of TfR1-mediated iron uptake pathway. Usually, Tf–TfR1 complex recycles in early endosomal pathway, whereas PV is formed after late endosomal pathway (McConville *et al*., 2007). So, the finding of Tf in PV was unusual although infection for 7 days might change in host–parasite interaction substantially including host cytoskeleton structures, which may allow the Tf to reach PV. In contrary, we detected a very fast depletion of LIP within a couple of hours of infection (Fig. 4). Considering doubling time of intracellular *Leishmania* as about 8–10 h (Ismaeel *et al*., 1998), provision of LIP as primary iron source seems more logical. That LIP is the primary source of iron for intracellular LD could be further inferred from the fact that chelation of LIP by iron chelator DFO inhibited growth of intracellular LD by about 75% (Fig. 6D). Another possible and rich iron source for pathogens residing in macrophages is available haeme from senescent red blood cell taken up through endocytosis pathway (Theurl *et al*., 2005). Because of their inability to synthesize haeme *Leishmania* parasites have to depend on external source for acquisition of this important prosthetic group, so for them TfR1-mediated pathway should be the preferred source of iron. To avail haeme, *Leishmania* actually protect and endocytose haemoglobin, which they acquire by a specific receptor (Krishnamurthy *et al*., 2005).

Finally, our study opens the possibility of finding a siderophore-like iron-scavenging mechanism in this trypanosomatid parasite. Several bacteria use iron complexed to siderophores that effectively compete with host iron proteins for iron (Braun and Killmann, 1999). A previous study ruled out the possibility of a secreted iron chelator from *L. chagasi* promastigote (Wilson *et al*., 1994). In our study, incubation of macrophages with media from LD promastigote showed no effect on LIP or TfR1 (data not shown), confirming the previous finding that promastigote does not secrete any iron chelator. Recently, a ZIP family ferrous iron transporter LIT-1 was identified in *L. amazonensis*, which was found to be essential for intracellular replication of the parasite (Huynh *et al*., 2006). Incidentally, the expression of LIT-1 was only detected in amastigote form, indicating that a new set of iron-scavenging and utilization machinery is expressed after the entry of parasite into macrophages. So, one can presume that like LIT-1, virulent LD also synthesizes/secretes iron scavenger after immediate entry into macrophage. The understanding of this iron-scavenging machinery might be helpful to find novel drug target for this intracellular pathogen for which available drugs are mostly resistant.

## Experimental procedures

Unless otherwise stated, all reagents were obtained from Sigma Chemical. Tissue culture supplies were obtained from Corning. TfR1 antibody was from Zymed, LAMP-1 and α-actin antibodies were obtained from Santa Cruz Biotechnology (Santa Cruz, CA). IRP1 and IRP2 antibodies were obtained from Santa Cruz Biotechnology as well as from Alpha Diagnostic International. HIF-1α was obtained from Novus Biologicals. All the secondary antibodies labelled with horseradish peroxidase were purchased from Bio-Rad.

### Animals

BALB/c female mice 4–12 weeks old (National Centre for Laboratory Animal Sciences, Hyderabad, India) were used for the experiments. All the experiments involving animals were performed as approved by the Institutional Animal Ethics Committee.

### Isolation and maintenance of spleen-derived macrophages and cell Lines

Splenic macrophages were isolated from BALB/c female mice following the procedure mentioned before (Wigley *et al*., 2001) and cultured in RPMI-1640 medium supplemented with 10% heat-inactivated fetal bovine serum (Hyclone), 100 units ml^−1^ penicillin and 100 μg ml^−1^ streptomycin. Mouse macrophage cell line J774A.1 (ATCC) was also cultured in the same medium. Cells were maintained in a humidified atmosphere containing 5% CO_2_ at 37°C in a CO_2_ incubator (Heraeus).

### Parasite culture

The LD (MHOM\IN\ 1983\AG83) was used for all the experiments and *L. major* (Friedlin) was also used (both are kindly provided by Prof. R. Madhubala). Culture and maintenance of the virulent parasites by passaging through BALB/c mice were performed as described before (Mukhopadhyay *et al*., 2000). Freshly transformed promastigotes were maintained in M199 medium supplemented with 10% FBS, 100 units ml^−1^ penicillin, 100 μg ml^−1^ streptomycin at 22°C. On every fourth to fifth day subculturing was performed after promastigotes attained stationary phase of growth.

### Immunoblot analyses

To determine effect of LD infection on TfR1 expression cell lysates were prepared after the requisite time of infection in buffer containing 50 mM Hepes, pH 7.5, 150 mM NaCl, 1 mM EDTA, 2 mM sodium vanadate, 1 mM phenylmthylsulfonyl fluoride, NP40 and a protease inhibitor cocktail (Roche) and proteins were measured by Bio-Rad assay Kit. Cell lysates (30 μg) were subjected to SDS-PAGE (7.5%) and transferred to PVDF membrane (Millipore). PVDF membranes were incubated with TfR1 antibody (1:2500). To determine the IRP1 and IRP2 contents, cell lysates (40 μg) were resolved in SDS-PAGE (8%) in reducing condition. Immmunoblotting was performed using IRP1 or IRP2 antibody (1:200). Protein bands were visualized by ECL (Amersham).

### Northern analysis and semiquantitative RT-PCR

Total RNA was isolated using TriPure reagent (Roche) and 20 μg of RNA was denatured in formamide/formaldehyde, electrophoresed through 1% agarose gel containing 6% formaldehyde, and blotted onto nylon membranes. After cross-linking filters were hybridized to an 890 bp fragment of mouse TfR1 (generated by RT-PCR and confirmed by sequencing) and labelled by random priming with [α-^32^P]-dCTP using a New England Biolab Kit.

The RT-PCRs were performed using one-tube RT-PCR system (Roche). For TfR1 following primers were used: forward, 5′-gct tga aga tcg tta g-3′ and reverse, 5′-cta aca cag taa agg tc-3′. Similarly, for mouse β-actin: forward, 5′-gac atg gag aag atc-3′ and reverse, 5′-gaa tgt agt ttc atg-3′ were used.

### TfR1 mRNA stability

Actinomycin D (5 μg ml^−1^) was added in the LD-infected and uninfected J774A.1 cells and total RNA were isolated at 0, 1, 2 and 3 h of actinomycin D addition. RT-PCR was performed with mouse-specific TfR1 primers as described previously and was run into 1% agarose gel to assess the mRNA stability. Similarly, mouse-specific β-actin primers were used for performing RT-PCR from the same RNA.

### Preparations of cytosolic extracts

Cytosolic extracts were prepared from J774A.1 cells and macrophages isolated from spleens of LD-infected and uninfected mouse as described earlier (Schneider and Leibold, 2003). Briefly, cells were treated with 0.01% digitonin for 15 min, scraped and spun at 600 *g* for 5 min at 4°C to pellet cells. The supernatant was removed and spun at 15000 *g* for 10 min at 4°C. The cleared lysate was saved as cytosolic fraction.

### Aconitase assay

Aconitase assay was performed as described previously (Drapier and Hibbs, 1986). Cytosolic extracts (50 μg) from uninfected, LD-infected or DFO (100 μM)-treated J774A.1 cells (12 h) were added to 0.2 mM *cis*-aconitate in 100 mM Tris-Cl, pH 7.4, 100 mM NaCl, BSA (0.02%) to perform aconitase assay as disappearance of *cis*-aconitate was followed at 25°C at 240 nm.

### Cloning of IRE containing mouse TfR1 3′UTR

A 188 bp fragment containing the proximal two IREs of the total five from 3′UTR of mouse TfR1 was PCR-amplified from genomic DNA by using following primers: forward, 5′-cag gaa agc ttt cta tc-3′ and reverse, 5′-ggg tct cat tag cag-3, cloned into pcDNA3 between BamHI and EcoRI sites under the control of T7 promoter and the clone was confirmed by sequencing.

### In vitro transcription and RNA-gel shift assay

pcDNA3 plasmid construct containing 188-nucleotide TfR1 3′UTR including two proximal IREs was linearized and transcribed using *in vitro* transcription kit (Roche). Using [α-^32^P]-UTP-labelled TfR1 3′UTR, RNA gel shift assays were performed following protocols as described before (Leibold and Munro, 1988). For the binding assays, 10 μg of cytoplasmic extracts was incubated with radiolabelled TfR1-3′UTR-IRE probe. For supershift analysis 2 μg of IRP1, IRP2 or HIF-1α antibody was added with cytosolic extract for 30 min before the addition of radiolabelled probe. RNA–protein complexes were resolved by 5% non-denaturing polyacrylamide gels. The gels were dried and subjected to autoradiography.

### Calcein-sensitive LIP assessment

Labile iron pool was assessed using Calcein-AM as described earlier (Stäubli and Boelsterli, 1998). In short, J774A.1 cells were infected with LD or treated with DFO (100 μM) as described previously and after the infection/treatment cells were washed with ice-cold PBS and kept in RPMI-1640 (without phenol red). Then, calcein-AM (0.5 μM) was added and the cells were incubated at 37°C for 20 min. Fluorescence microscopy was done at 488 nm excitation and 517 nm emission. Nikon upright fluorescence microscope model 80i equipped with water emersion objectives and connected with cooled CCD digital camera was used for imaging.

### Radiolabelled iron uptake assay

Initially, Apo-Tf was loaded with radioactive iron as described earlier (Klausner *et al*., 1983). Briefly, ^55^FeCl_3_ (Perkin Elmer) and nitrilotriacetate (NTA) were mixed in 1:50 molar ratio in 300 μl of carbonate buffer (10 mM NaHCO_3_, 250 mM Tris-Cl pH 7.4). After mixing thoroughly, it was kept at room temperature for 10–15 min. Apo-Tf was incubated with ^55^Fe–NTA in 1:2 ratios at room temperature for 1 h and then kept at 4°C overnight. The complex was passed through G-50 sephadex column for purifying the ^55^Fe–Tf complex from the unbound ^55^Fe–NTA.

To measure iron uptake 1 × 10^5^ J774A.1 cells were infected with LD at a ratio of 1:10 or treated with DFO (100 μM). Then, cells were washed with serum-free RPMI twice and ^55^Fe–Tf was added in serum-free RPMI. After 30 min, the cells were washed with 150 mM NaCl with 100 μM EDTA solution twice to remove extracellularly bound ^55^Fe–Tf. The cells were then lysed in 200 μl of lysis buffer (0.1% NaOH and 0.1% TritonX-100) at 4°C. The radioactive iron was measured in cell lysate using liquid scintillation counter (Beckman LS6500).

### Intracellular amastigote counting

The intracellular amastigotes from J774A.1 cells were counted using percoll gradient as described earlier (Chang, 1980). Briefly, infected macrophages were lysed by four freeze–thaw cycles. Then cell lysates were put in an individual percoll gradient (in the order of 90%, 40% and 20%) and spun at 800 *g* for 1 h. The band at the interface of 90%/40% percoll is collected and the volume of each collection is equilibrated up to 1 ml. Parasites were counted in the improved Neubaur Counting Chamber.
